# Hepatic Injury Induced by Dietary Energy Level via Lipid Accumulation and Changed Metabolites in Growing Semi-Fine Wool Sheep

**DOI:** 10.3389/fvets.2021.745078

**Published:** 2021-09-23

**Authors:** Benchu Xue, Qionghua Hong, Xiang Li, Mingli Lu, Jia Zhou, Shuangming Yue, Zhisheng Wang, Lizhi Wang, Quanhui Peng, Bai Xue

**Affiliations:** ^1^Animal Nutrition Institute, Sichuan Agricultural University, Chengdu, China; ^2^Yunna Academy of Animal Science and Vetarinary Medicine, Kunming, China; ^3^Department of Bioengineering, Sichuan Water Conservancy College, Chengdu, China

**Keywords:** liver, injury, metabonomics, energy level, oxidative stress

## Abstract

Liver injury threatens the overall health of an organism, as it is the core organ of the animal body. Liver metabolism is affected by numerous factors, with dietary energy level being a crucial one. Therefore, the present study aimed to evaluate hepatic injury and to describe its metabolic mechanism in ruminants fed diets with different dietary energy levels. A total of 25 Yunnan semi-fine wool sheep were fed diets with five dietary metabolic energy levels and were randomly assigned to five groups as follows: low energy (LE), medium–low energy (MLE), medium energy (ME), medium–high energy (MHE), and high energy (HE). The results revealed that the average optical density (AOD) of lipid droplets in the LE, MLE, and HE groups was higher than that in the ME and MHE groups. The enzyme activity of alanine aminotransferase (ALT) was the lowest in the ME group. An increase in dietary energy level promoted the superoxide dismutase (SOD) and glutathione peroxidase (GSH-Px) activity and altered the malondialdehyde (MDA) and protein carbonyl (PCO) concentration quadratically. In addition, both high and low dietary energy levels upregulated the mRNA abundance of proinflammatory cytokine interleukin (IL)-1β, nuclear factor-kappa B (NF-κB), and tumor necrosis factor (TNF)-α. Metabonomic analysis revealed that 142, 77, 65, and 108 differential metabolites were detected in the LE, MLE, MHE, and HE groups, compared with ME group respectively. These metabolites were involved in various biochemical pathways, such as glycolipid, bile acid, and lipid metabolism. In conclusion, both high and low dietary energy levels caused hepatic injury. Section staining and metabonomic results revealed that hepatic injury might be caused by altered metabolism and lipid accumulation induced by lipid mobilization.

## Introduction

Yunnan semi-fine wool sheep, characterized by optimal reproductive performance, stable genetic performance, strong adaptability, and excellent meat performance and shearing capacity, is an important breed in the development of China's livestock industry (1). In sheep breeding, the focus is often on growth performance, and hence, the health of the heart, lungs, kidneys, spleen, liver, and other organs is usually ignored. Organ health is an important factor that not only determines whether the lamb can develop quickly but also reflects its overall health (2). The liver is the most important metabolic organ, with functions such as bile secretion, glycogen storage, and the regulation of protein, fat, and carbohydrate metabolism.

Several studies have reported that dietary energy concentration exerts an important effect on the development of ruminant organs, with the liver being the most significantly affected (3). However, with an increase in energy intake, the liver may also be affected by simultaneous damage, including liver function impairment and oxidative damage (4, 5), which may subsequently aggravate hepatocyte apoptosis and inflammatory response. Researchers have reported that fatty liver causes hepatic injury (6, 7). Fatty liver is a reversible disease, with large amounts of triglycerides or lipid droplets accumulating in hepatocytes through steatosis, which adversely affects the development, health, and reproduction of cows (8, 9). Diets with high energy levels downregulated the expression of 5′ adenosine monophosphate-activated protein kinase (AMPK) signaling pathways, thereby enhancing the expression of lipid synthesis-related genes, promoting lipid synthesis in the liver cells, reducing lipid oxidation, and increasing the triglyceride concentration (10, 11). However, hepatic lipid accumulation in animals fed diets with low energy levels has not been reported yet. Studies on fatty liver disease in dairy cows have reported a similar insufficient energy intake condition, which suggests that lipid accumulation is frequently observed in the liver of animals fed diets with low energy levels (12). Studies on dietary energy levels that affect hepatic health are now mostly focused on poultry (13, 14). The systematic evaluation of hepatic health is overlooked when ruminants are fed diets with different dietary energy levels. Therefore, it is necessary to systematically assess the effects of dietary energy levels on hepatic health in Yunnan semi-fine wool sheep to understand their metabolism.

Metabolomics displays all small-molecule metabolites produced by alterations in the nutritional status of an organism, thereby providing a more comprehensive and direct insight into the chemical processes and changes in nutritional status (15). Ippolito et al. used gas chromatography–mass spectrometry (GC-MS) to analyze changes in the plasma nutrient metabolome of rats under heat stress and obtained 28 heat stress markers, involving those for apoptosis or catabolism, altered energy balance, and cholesterol and nitric oxide metabolism (16). Zhang et al. employed nuclear magnetic resonance (NMR) for analyzing the plasma of cows exhibiting postpartum estrus, which showed that the levels of seven plasma metabolites were significantly lower in the estrus period than those in the normal period, demonstrating that estrus is accompanied by altered amino acid, glucose, and lipid metabolism (17).

Various studies have been reported on the energy requirements of the sheep. However, only a few studies have focused on the local breed of Yunnan semi-fine hair sheep. Li reported that the metabolic energy requirement of the sheep was 0.4359 MJ/kg of body weight (BW) 0.75/day + 0.0387 average daily gain (ADG) (18). Based on her study, we designed a trial to investigate hepatic health and metabolism by controlling the metabolic energy level of the diet using the metabolomic technique liquid chromatography with tandem MS (LC-MS/MS) in combination with multivariate statistical analysis. The objective of this study was to evaluate the injury caused by dietary energy levels, identify an energy level that is the most beneficial for the hepatic health of Yunnan semi-fine hair sheep, and investigate its metabolic mechanism.

## Materials and Methods

### Experimental Design, Animals, and Sample Collection

All experimental protocols were approved by the Animal Ethical and Welfare Committee (AEWC) of the Sichuan Agricultural University Academy of Sciences (approval no. 20180601). This study was conducted in accordance with the Chinese Guidelines for Animal Welfare. This study was conducted at the farm of the Sichuan Agricultural University (Ya'an, Sichuan Province, China). In this study, a total of 25 Yunnan semi-fine hair wether sheep, with similar BW (33.30 ± 1.77 kg), were randomly assigned to five groups. Each group of sheep was reared in a pen. The sheep in the five groups were fed diets with five metabolic energy levels as follows: 8.0, 8.6, 9.2, 9.8, and 10.4 MJ/kg. According to the standard energy requirement of Yunnan semi-fine hair sheep (BW: 33.3 kg and ADG: 80 g/day) (18), these five groups were distinguished as follows: low energy (LE; 86% energy requirement), medium–low energy (MLE; 93% energy requirement), medium energy (ME; 100% energy requirement), medium–high energy (MHE; 107% energy requirement), and high energy (HE; 114% energy requirement). The ingredients and chemical compositions of each diet are provided in Table 1.

**Table 1:** Composition and nutrient levels of experimental diets.

**Items**	**Groups**				
	**LE^c^**	**LME**	**ME**	**MHE**	**HE**
Ingredients	Content (%)				
Corn	11.00	19.35	28.15	34.15	30.00
Wheat bran	26.15	16.80	7.00	0.00	0.00
Soybean meal	6.00	7.00	8.00	9.00	9.00
Corn starch	0.00	0.00	0.00	0.00	4.15
NaCl	0.50	0.50	0.50	0.50	0.50
NaHCO_3_	0.35	0.35	0.35	0.35	0.35
Premix^a^	1.00	1.00	1.00	1.00	1.00
Corn silage	10.00	21.00	33.00	40.00	55.00
Wheat straw	45.00	34.00	22.00	15.00	0.00
Total	100	100	100	100	100
Concentrate : roughage nutrient levels^b^	45:55	45:55	45:55	45:55	45:55
ME (MJ/kg)	8.00	8.60	9.20	9.80	10.40
Dry matter (%)	92.62	92.19	91.88	91.47	91.20
CP (%)	10.42	10.42	10.42	10.48	10.46
NDF (%)	53.80	46.71	39.10	34.24	28.10
ADF (%)	21.96	20.92	19.62	19.63	19.20
Ca (%)	0.42	0.40	0.37	0.35	0.35
P (%)	0.44	0.38	0.33	0.39	0.37

a *One kilogram of premix contained the following: VA 500,000 IU, VD_3_ 200,000 IU, VE 850 IU, nicotinic acid 1.2 g, Cu 2 g, Fe 10 g, Zn 6 g, Mn 5 g, I 100 mg, Co 55 mg, Se 35 mg*.

b *ME was a calculated value, and others were measured values*.

c *LE, MLE, ME, MHE, and HE represent the following groups: low energy, medium–low energy, medium energy, medium–high energy, and high energy, respectively*.

The trial lasted 45 days, with the first 15 days being the preliminary period for the sheep to adapt to the diets and 30 days being the formal trial period. The sheep were fed twice daily at 8:00 AM and 6:00 PM and had free access to water. The criteria for euthanizing the sheep before the experiment included anesthesia and neck bleeding.

On the 30th day of the formal trial, three sheep from each group were selected to be weighed and anesthetized, which were eventually euthanized. Subsequently, the whole liver was separated and weighed. The left liver was collected and stored in 4% paraformaldehyde and PBS for liver section staining. The right liver was collected in a 30-ml Eppendorf (EP) tube for later measurement.

### Weight of the Liver and Liver Index

The data for the final BW and liver weight (LW) of sheep were recorded, and the liver index (LI) was calculated as LI = LW/BW (%).

### Determination of Hepatic Lipid Accumulation

Liver tissues were fixed with 10% formalin; they were embedded, sliced, and stained with Oil Red O. A microscopic imaging system was used to capture pictures. A total of three pictures were captured for each sample. Image-Pro Plus 6.0 software was used for analyzing the average optical density (AOD) of the positive results of Oil Red O staining.

### Determination of Transaminase and Oxidative Damage

The enzyme activity of alanine aminotransferase (ALT), aspartate aminotransferase (AST), malondialdehyde (MDA), protein carbonyl (PCO), superoxide dismutase (SOD), catalase (CAT), and glutathione peroxidase (GSH-Px) and the total antioxidant capacity (T-AOC) of liver samples were determined using enzyme-linked immunosorbent assay (ELISA) kits (19), purchased from Jiangsu Meimian Industrial Co., Ltd (Jiangsu, China).

### Real-Time Polymerase Chain Reaction of Hepatic Inflammation-Related Genes

Inflammation-related gene expression was determined using reverse-transcription polymerase chain reaction (RT-PCR). Total RNA was extracted from the liver tissues using the TRIzol RNA kit (Takara, Japan), catalog no. 15596026. Then its purity and content were measured. cDNA was obtained through reverse transcription of RNA samples with the TaKaRa Reverse Transcription Kit. Real-time PCR (qPCR) primers were designed using Primer 3 online software (Supplementary Table 1), and gene expression was determined using an ABI 7900 fluorescent quantitative PCR instrument using the SYBR (Takara) dye. The reaction conditions were as follows: 95°C for 30 s, followed by 40 cycles of amplification (95°C for 5 s and 60°C for 34 s) with melting phase set to 95°C for 15 s, 60°C for 1 min, and 95°C for 15 s. Glyceraldehyde 3-phosphate dehydrogenase (GAPDH) was used as an internal reference gene, and its expression level was analyzed using the 2^−ΔΔCt^ method.

### Untargeted Metabonomics and Its Analysis

Samples for metabolites were extracted from liver tissues (20). LC-MS/MS analyses were performed using a Vanquish UHPLC system (Thermo Fisher Scientific) coupled with an Orbitrap Q Exactive HF-X mass spectrometer (Thermo Fisher Scientific) in both positive and negative modes. The raw data files generated by UHPLC-MS/MS were processed using the Compound Discoverer 3.1 (CD3.1, Thermo Fisher) to perform peak alignment, peak picking, and quantitation for each metabolite.

Principal component analysis (PCA) and partial least squares discriminant analysis (PLS-DA) were performed at metaX (a flexible and comprehensive software for processing metabolomics data). Variable importance in the projection (VIP) is the importance of the variables to the model and describes the overall contribution of each variable to the differences between groups. We applied univariate analysis (*t*-test) to calculate the statistical significance (*p*-value). The metabolites with a VIP > 1, *p*-value < 0.05, and fold change (FC) ≥2 or ≤0.5 were considered to be differential metabolites.

As differential metabolites were selected, the metabolic pathways for these differential metabolites were obtained from the Kyoto Encyclopedia of Genes and Genomes database (http://www.genome.jp/kegg).

### Statistical Analysis

Statistical analyses except metabonomics were performed using the one-way ANOVA procedure of the SPSS statistical software (version 17.0, SPSS Inc., Chicago, IL, USA). Duncan's multiple-range test was used to compare the differences among five groups. The data were expressed as mean ± SEM. A *p*-value <0.05 was set for significant differences. A value of 0.05 < *p* < 0.10 was set for the trend of significant differences.

## Results

### Liver Weight and Index

The LW and LI of Yunnan semi-fine wool sheep are provided in Table 2. With an increase in dietary energy levels, LW and LI of the sheep were increased and subsequently stabilized (*p* < 0.05). There were no significant differences among HE, MHE, and HE groups.

**Table 2:** The effects of dietary energy level on liver weight and index of Yunnan semi-fine wool sheep.

**Items**	**Energy level**					**SEM**	***P*** **-value**		
	**LE**	**MLE**	**ME**	**MHE**	**HE**		**Energy**	**Linear**	**Quadratic**
Liver Weight (g)	471.93^c^	534.30^b^	603.87^a^	600.93^a^	608.97^a^	15.68	0.001	<0.001	0.017
Body Weight (kg)	34.90^c^	36.69^bc^	39.50^ab^	39.16^ab^	40.13^a^	0.62	0.007	0.001	0.169
Liver Index (%)	1.35^b^	1.45^ab^	1.53^a^	1.54^a^	1.52^a^	0.025	0.079	0.017	0.099

*Values with different letters were significantly different (p < 0.05)*.

### Hepatic Lipid Accumulation

**Figure 1:**
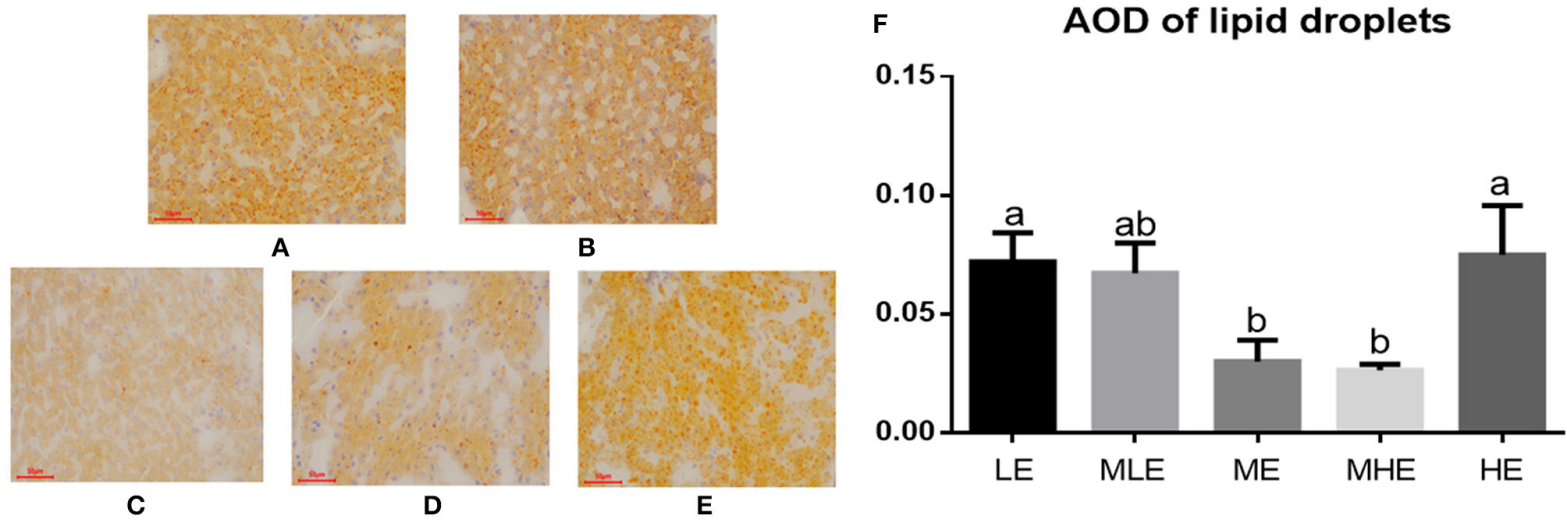
The effects of dietary energy level on hepatic lipid accumulation of Yunnan semi-fine wool sheep. The results of Oil Red O staining for LE **(A)**, MLE **(B)**, ME **(C)**, MHE **(D)**, HE **(E)** groups. The AOD values **(F)**. Values with different letters were significantly different (*p* < 0.05).

The results of Oil Red O staining of the liver are provided in Figures 1A–E. Lipid droplets in hepatic cells were represented in an orange-red color (bar = 50 μm, × 200). The concentration of lipid droplets was higher in the LE, MLE, and HE groups than in the ME and MHE groups. The quantitative analysis of AOD values (Figure 1F) revealed that dietary energy levels had a significant effect on the AOD of liver lipid droplets (*p* < 0.05), and the AOD values of LE and HE groups were significantly higher than those of the ME and MHE groups.

### Assessment of Hepatic Injury

The assessment of hepatic injury was based on transaminase activity, oxidative damage, and inflammation.

**Figure 2:**
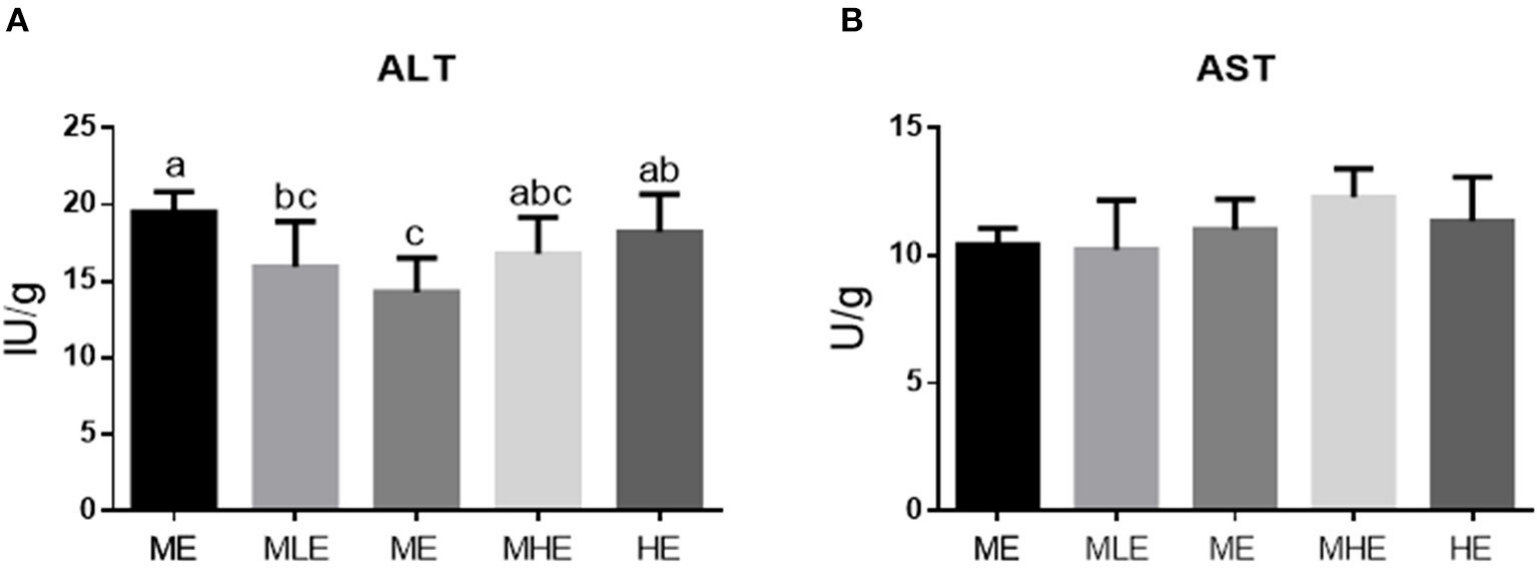
The effects of dietary energy level on transaminase enzyme activity of Yunnan semi-fine wool sheep. The result of ALT **(A)**. The result of AST **(B)**. Values with different letters were significantly different (*p* < 0.05).

The assessment of transaminase activity in the liver is provided in Figure 2. The ALT enzyme activity in the liver decreased and subsequently increased with an increase in dietary energy levels (*p* < 0.05). The ALT enzyme activity of the ME group was significantly lower than that of the LE and HE groups (*p* < 0.05; Figure 2A). Dietary energy levels had no significant effect on the liver AST enzyme activity (*p* > 0.05; Figure 2B).

The assessment of oxidative injury in the liver is provided in Table 3. With an increase in dietary energy levels, the SOD and GSH-Px enzyme activities increased linearly (*p* < 0.05). MDA concentration in the ME and MHE groups was significantly lower than that in the remaining three groups (*p* < 0.05). PCO concentration in the ME group was significantly lower than that in the HE group (*p* < 0.05). CAT concentrations in the LE and ME groups were significantly higher than those in the remaining three groups (*p* < 0.05).

**Table 3:** The effects of dietary energy level on oxidative injury of Yunnan semi-fine wool sheep.

**Items**	**Energy level**					**SEM**	***P*** **-value**		
	**LE**	**MLE**	**ME**	**MHE**	**HE**		**Energy**	**Linear**	**Quadratic**
MDA (nmol/L)	11.57^a^	11.91^a^	9.10^b^	8.84^b^	12.67^a^	0.39	<0.001	0.088	0.952
PCO (pg/ml)	189.80^ab^	187.51^ab^	154.97^b^	185.80^ab^	210.92^a^	5.79	0.035	0.224	0.010
SOD (U/ml)	70.79^c^	85.14^b^	91.87^ab^	96.39^ab^	105.58^a^	2.92	<0.001	<0.001	0.482
CAT (U/ml)	20.68^a^	16.54^c^	21.74^a^	19.33^ab^	17.11^bc^	0.51	<0.001	0.110	0.236
GSH-Px (U/L)	150.67^c^	167.00^bc^	192.91^ab^	189.87^ab^	209.43^a^	6.21	0.014	0.001	0.613
T-AOC (U/ml)	6.26	7.46	5.65	6.53	7.34	0.26	0.164	0.497	0.380

*MDA, Malondialdehyde; PCO, protein carbonylation; SOD, superoxide dismutase; CAT, catalase; GSH-Px, glutathione peroxidase; T-AOC, total antioxidant capacity. Values with different letters were significantly different (p < 0.05)*.

**Figure 3:**
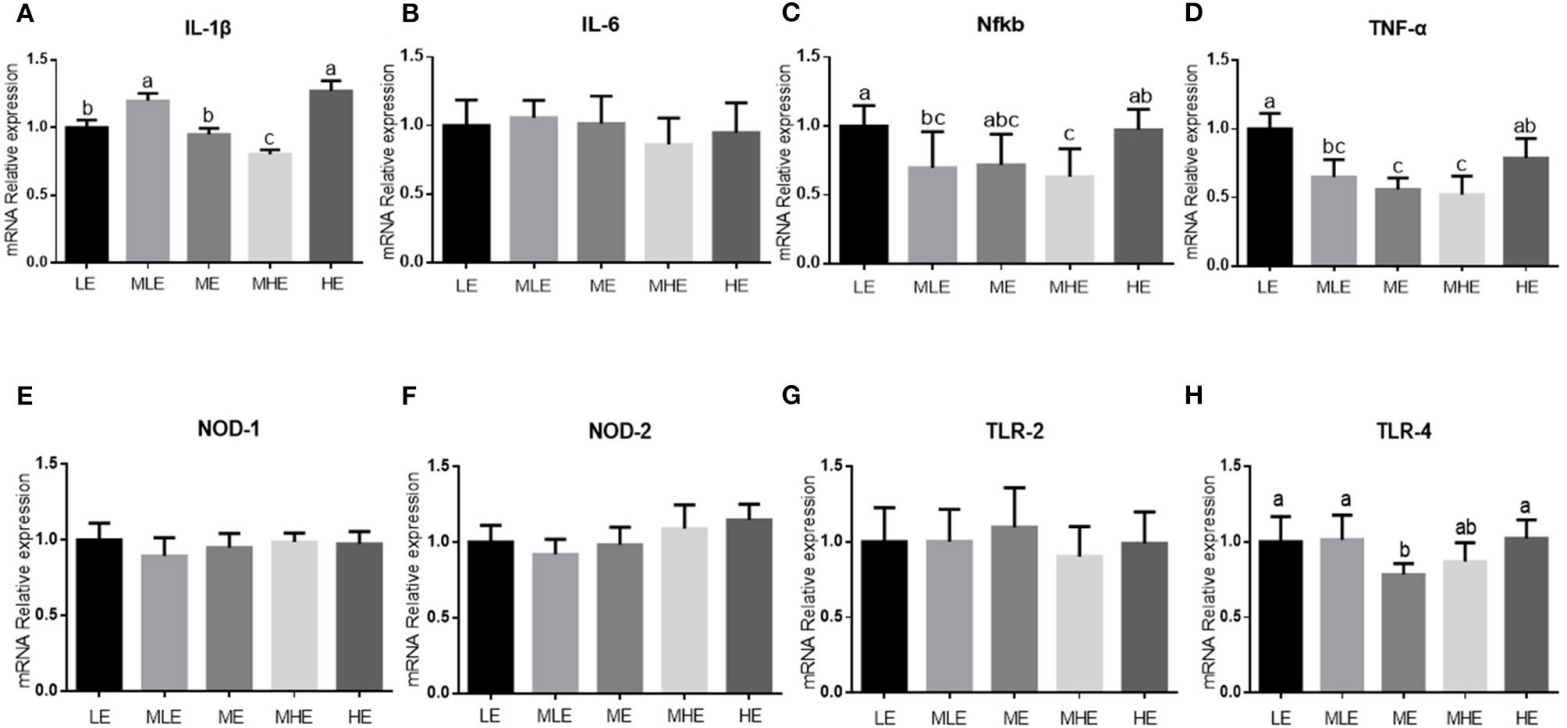
The effects of dietary energy level on expression of hepatic genes related to inflammation in Yunnan semi-fine wool sheep. Real-time RT-PCR analysis for mRNA expression of hepatic genes including **(A)** IL-1β; **(B)** IL-6; **(C)** NFKB; **(D)** nucleotide-binding oligomerization domain 2 (NOD2); **(E)** Toll-like receptors 2 (TLR2); **(F)** Toll-like receptors 3 (TLR3); **(G)** Toll-like receptors 4 (TLR4); and **(H)** TNF-α. Values with different letters were significantly different (*p* < 0.05).

The assessment of the relative expression of inflammation-related genes in the liver is provided in Figure 3. With an increase in dietary energy levels, the expression of toll-like receptor-2 (*TLR-2*) and tumor necrosis factor-α (*TNF-*α) in the liver was downregulated and subsequently upregulated (*p* < 0.05; Figures 3F,H). The expression of interleukin-6 (*IL-6*) gene in the liver was upregulated in the MLE and HE groups, followed by LE, ME, and MHE groups (*p* < 0.05; Figure 3B). The dietary energy level had no significant effect on the expression of IL-6, nuclear factor-kappa B (*NFKB*), *TLR-3, TLR-4*, and nucleotide-binding oligomerization domain-containing protein 2 (*NOD-2*) in the liver (*p* > 0.05; Figures 3C–E,G).

### Metabolomic Profiling of Liver Samples

As demonstrated in Supplementary Figure 1, the overlap degree of total ion chromatography (TIC) of quality control (QC) samples was significantly high in both positive (Supplementary Figure 1A) and negative (Supplementary Figure 1B) modes, indicating that the LC-MS/MS system was highly stable.

**Figure 4:**
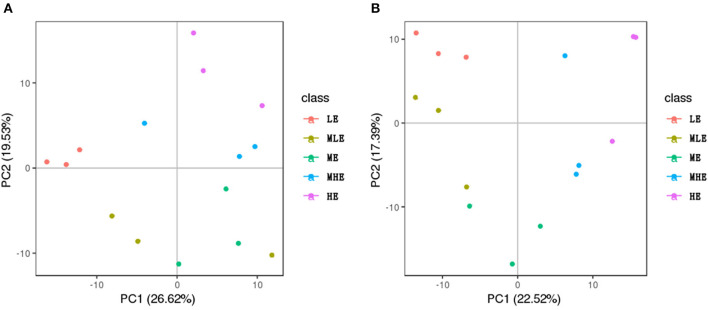
The PCA score plots based on the liver metabolic profiling. **(A)** Positive mode. **(B)** Negative mode.

All liver samples were subjected to PCA as demonstrated in Figure 4. In the negative ion mode (Figure 4A), the five groups were separated from each other, and in the positive mode (Figure 4B), the five groups were separated more clearly.

PLS-DA was performed to analyze the differences among the groups. The LE, MLE, MHE, and HE groups were compared with the ME group for PLS-DA. The results of PLS-DA in the positive and negative modes are provided in Supplementary Figures 2, 3, respectively.

### Identification of Different Metabolites

**Figure 5:**
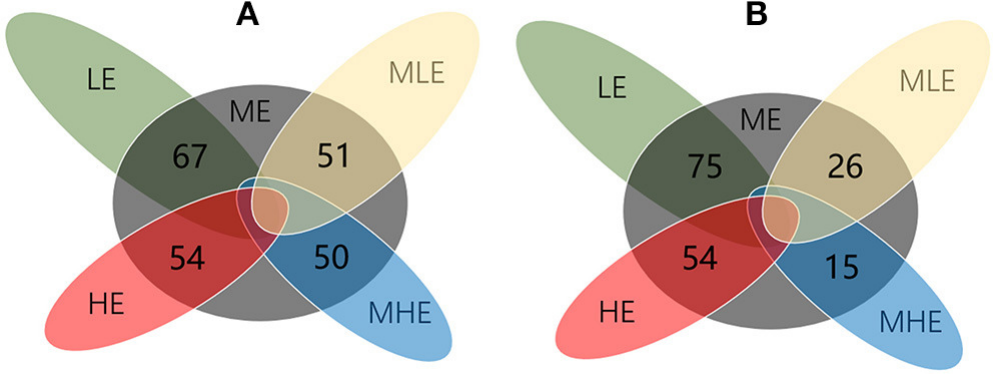
The Venn diagram for illustrating the number of differential metabolites among groups. **(A)** Positive mode. **(B)** Negative mode.

Compared with the ME group, 67, 51, 50, and 54 differential metabolites were detected in the LE, MLE, MHE, and HE groups, respectively, in the positive mode (Figure 5A). Similarly, 75, 26, 15, and 54 differential metabolites were detected in the LE, MLE, MHE, HE, and ME groups, respectively, in the negative mode (Figure 5B). The metabolites related to hepatic metabolism and health are listed in Table 4.

**Table 4:** Identification of different metabolites in the liver.

**Metabolites**	**LE vs. ME**			**MLE vs. ME**			**ME vs. MHE**			**ME vs. HE**		
	**P**	**VIP**	**FC**	**P**	**VIP**	**FC**	**P**	**VIP**	**FC**	**P**	**VIP**	**FC**
6-Phosphogluconic acid	<0.001	1.34	0.12	0.048	1.05	4.21	–	–	–	–	–	–
d-Glucose 6-phosphate	–	–	–	0.002	1.25	1.65	0.003	1.23	0.65	0.011	2.29	0.33
d-Erythrose 4-phosphate	–	–	–	–	–	–	–	–	–	0.004	2.16	0.47
*N*-Acetyl-d-galactosamine	<0.001	2.21	0.2	0.002	1.3	0.41	–	–	–	0.001	1.54	3.36
d-Sedoheptulose-7-phosphate	–	–	–	0.013	2.34	1.77	–	–	–	0.019	1.54	0.64
Citraconic acid	0.035	1.46	1.52	–	–	–	–	–	–	0.017	1.83	0.59
Citric acid	0.038	1.39	1.53	0.042	1.88	1.62	–	–	–	–	–	–
l-Asparagine	–	–	–	–	–	–	–	–	–	0.018	1.69	0.63
dl-Malic acid	0.009	1.67	1.77	0.034	1.49	1.53	–	–	–	–	–	–
Hexanoic acid	0.035	1.62	2.03	–	–	–	–	–	–	0.024	1.67	0.48
Acetoacetate	0.002	1.77	1.68	–	–	–	–	–	–	0.001	2.1	0.55
Oxoadipic acid	0.006	2.03	2.25	–	–	–	–	–	–	0.032	1.08	0.66
Uridine	0.008	1.53	0.48	0.035	1.17	0.63	0.031	2.54	2.37	0.006	1.7	2.26
d-Ribose	0.017	1.59	1.88	0.017	1.93	1.89	0.039	1.9	0.58	0.023	1.89	0.47
Ala–leu	0.038	1.61	2.4	–	–	–	0.026	1.77	0.44	–	–	–
Adenylosuccinic acid	0.05	1.38	1.5	0.048	1.71	1.53	–	–	–	–	–	–
Glutaric acid	0.011	1.54	1.58	–	–	–	–	–	–	0.006	2.12	0.53
dl-Glutamine	0.028	1.58	0.66	–	–	–	–	–	–	–	–	–
l-Glutathione	–	–	–	0.014	1.91	0.43	–	–	–	–	–	–
Cys-gly	0.027	1.4	3.07	–	–	–	–	–	–	0.04	1.24	0.38
Cystamine	0.005	1.74	2.73	0.008	1.56	2.35	–	–	–	0.003	1.86	0.31
Cystine	–	–	–	0.001	1.25	1.62	–	–	–	–	–	–
*S*-Adenosylmethionine	–	–	–	–	–	–	0.003	1.71	0.49	0.001	2.09	0.33
Taurine	–	–	–	–	–	–	–	–	–	0.002	1.93	0.5
Cholic acid	0.024	1.09	3.26	–	–	–	–	–	–	–	–	–
Taurodeoxycholic acid	0.019	1.25	0.19									
Taurochenodeoxycholic acid	0.027	1.23	0.46	–	–	–	–	–	–	–	–	–
Glycodeoxycholic acid							0.001	2.56	3.31	0.033	1.29	2.28
Hydrocortisone	0.008	1.18	0.2	–	–	–	–	–	–	–	–	–
*N*-acetylmannosamine	0.014	1.63	0.36	0.015	1.56	0.45	–	–	–	0.004	1.99	3.39
GDP	0.01	1.37	1.53	0.004	2.28	1.79	–	–	–	0	1.77	0.58
Acetyl phosphate	–	–	–	0.034	1.73	1.71	–	–	–	0.005	1.76	0.53
4-Methylphenol	–	–	–	0.018	2.13	0.5	0.045	1.83	1.69	0.034	1.79	1.96
3-Indoxyl sulfate	0.002	1.07	0.54	0.04	1.89	0.43	0.002	2.18	2.44	0.017	2.04	3.05
NAD+	–	–	–	–	–	–	0.012	1.27	0.65	0.001	2.07	0.41
Methylamino-l–alanine	0.001	2.22	0.22	–	–	–	0.013	1.14	1.93	–	–	–
l-ergothioneine	0.021	1.42	0.58	0.032	1.39	0.61	–	–	–	–	–	–
l-adrenaline	0.018	1.62	1.71	–	–	–	–	–	–	0.008	1.98	0.49
Glycolithocholic acid	0.017	1.7	0.51	0.005	2.17	0.45	0.005	2.5	2.26	0.014	1.65	2
Betaine	0.023	1.58	1.51	0.004	2.13	1.68	–	–	–	–	–	–
ADP	–	–	–	–	–	–	0.032	1.16	0.64	0.004	1.83	0.4
(5–l-glutamyl)-l-amino acid	0.01	1.72	2.12	0.006	2.14	2.41	0.017	1.96	0.48	0.021	1.71	0.44

*Ala–Leu, alanine—leucine; Cys–Gly, cysteine—glycine; GDP, guanosine diphosphate; NAD^+^, nicotinamide adenine dinucleotide*.

### Integration of Key Different Metabolic Pathways

**Figure 6:**
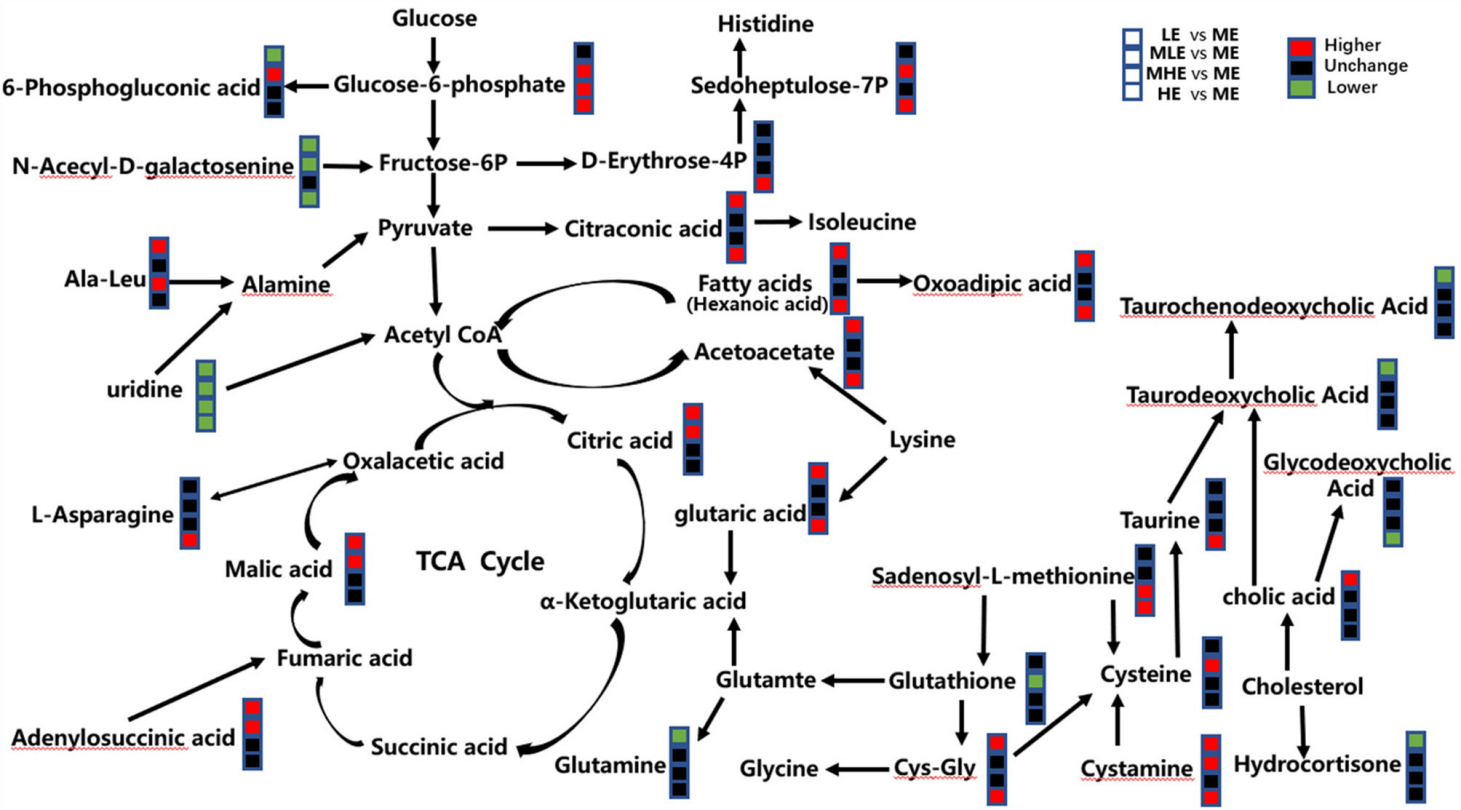
Different metabolic pathways from the sheep of five groups. The red mark means that the concentration of the former group is higher than that of the latter group. The black mark means the difference is not significant, and the green mark means the concentration of the former group is lower than that of the latter group.

The metabolic pathway analysis by the KEGG database and literature showed that these differential metabolites were involved in various biochemical pathways, such as amino acid metabolism, glycolipid metabolism, bile acid metabolism, nucleotide metabolism, and energy metabolism. In order to visualize the correlation between these metabolites, the results were finally combined, and a metabolic network diagram was drawn (Figure 6).

## Discussion

Organ tissues only constitute 6%−10% of BW; however, their energy consumption is as high as 50% of total energy in ruminants (21). Therefore, organ development requires energy, especially liver development (22). The organ weight and index can reflect organ development to a certain extent. As shown in Table 1, LW and LI were the highest in the ME group because excess energy may not promote liver development once the energy requirements have been fulfilled. However, an injury may also be caused by increased energy levels.

We found that dietary energy levels induced significantly different degrees of hepatic injury in semi-fine wool sheep. In particular, the enzyme activity of ALT varied quadratically with an increase in dietary energy level and was the lowest in the ME group. Because the enzyme activity of transaminase is an effective indicator of hepatic injury (23), both high and low dietary energy intakes may cause hepatic injury. With regard to oxidative injury, the effects of SOD and GSH-Px were similar, which increased with an increase in dietary energy levels. However, MDA and PCO concentrations demonstrated a quadratic trend similar to that of ALT, which is the peroxide product. These results indicated that the antioxidant function was increased with an increase in dietary energy levels; however, the liver exhibited more oxidative injury with high dietary energy levels. This may be because excessive nutrient intake and absorption aggravate the metabolism of the body, which inevitably produces products such as reactive oxygen radicals (24). Similar results were obtained in the expression of inflammation-related hepatic genes (TLR-4 and TNF-α). Owing to TLR signaling, a large number of inflammatory mediators such as IL-1β, IL-6, and TNF-α reacted on the organism and produced a series of inflammatory responses (25), demonstrating that sheep in the LE and HE groups were more likely to exhibit inflammation.

The liver is a core organ in the regulation of lipid metabolism. Limited production of very-low-density lipoproteins (VLDL) in ruminants results in low export of triglycerides. Delayed export of triglycerides produced by hepatocytes may lead to lipid deposition in the liver, thus triggering fatty liver and hepatic injury (26, 27). In this study, lipid deposition was reflected by the AOD of lipid droplets, which demonstrated significantly higher hepatic lipid deposition in the LE, MLE, and HE groups than that in the ME and MHE groups. Based on the AOD of liver lipid droplets, both low and high dietary energy levels have the potential to develop fatty liver disease. Mitochondria in cows with fatty liver produce more superoxide anions and hydrogen peroxide, leading to chronic oxidative stress (24), which is consistent with our assessment of oxidative injury. Therefore, hepatic injury may be induced by lipid accumulation.

Because hepatic injury is confirmed, hepatic lipid accumulation may be one of the causes. However, other factors may affect liver health as well. Therefore, we investigated differences in the global metabolic profiles of five dietary energy levels.

In this study, we employed LC-MS/MS to determine differences in the metabolic profiles of the liver. A huge number of differential metabolites were detected, which were involved in various biochemical pathways, such as amino acid metabolism, glycolipid metabolism, bile acid metabolism, nucleotide metabolism, and energy metabolism. We have described below the involvement of these pathways in hepatic injury.

### Glucose Metabolism

We found that the MHE and HE groups exhibited a significantly higher concentration of glucose 6-phosphate than that exhibited by the ME group. The concentration of *N*-acetyl-d-galactosamine (a precursor of fructose 6-phosphate) was significantly lower in the LE group than in the ME group, which was consistent with previous studies, suggesting that the glycolysis pathway is enhanced in the presence of adequate energy substrates (28). The concentration of glucose 6-phosphate was also significantly lower in the MLE group than in the ME group, which may be due to inflammation. The proinflammatory cytokines IL-6, TNF-α, and IL-1β were found to enhance glycolysis (29), which was consistent with the assessment of inflammation-related gene expression.

### Lipid Metabolism

The liver tissues of the LE, MLE, and HE groups exhibited more severe lipid deposition. Metabolomic results revealed that the concentration of hexanoic acid, as a free fatty acid (FFA), was significantly higher in the LE group than in the ME group, confirming that liver lipid deposition in the LE group may be caused by the reesterification of FFA in the liver owing to lipid mobilization, which results from insufficient energy intake. The assessment of acetoacetic acid, one of the intermediate metabolites of fatty acid oxidation in the liver (30), and acetic acid yielded similar results, suggesting that they supplied energy through fatty acid oxidation in the LE group. Owing to a sufficient energy intake in the HE group, the level of fatty acid oxidation in the liver should be lower (31), and lipid synthesis metabolism should be enhanced (32). As a result, lipid deposition was higher in the HE group than in the ME group. As amounts of very low-density lipoproteins (VLDLs) were restricted, their ability to export triglycerides is extremely limited. Therefore, sheep fed diets with both high and low energy levels are at a higher risk for developing fatty liver disease.

### Energy Metabolism

In this study, the three intermediate metabolites of the tricarboxylic acid cycle, namely, citric acid, malic acid, and adenylosuccinic acid (one of the precursors of ferredoxin), exhibited significantly higher concentrations in the LE group than that exhibited by the ME group, which is inconsistent with Wu's study (30). Combined with lipid metabolism, this may be due to the higher concentration of acetyl coenzyme generated by FFA β-oxidation in the LE and MLE groups.

Nicotinamide adenine dinucleotide (NAD+), guanosine diphosphate (GDP), and adenosine diphosphate (ADP) are involved in energy metabolism (33). The concentrations of NAD+, GDP, and ADP were significantly higher in the HE group than those in the ME group, which indicates that the sheep in the HE group were more likely to exhibit metabolic injury compared with those in the ME group owing to a high energy intake. High dietary energy levels significantly enhanced the levels of substrate energy metabolism and bio-oxidation (34, 35). In addition, it has been demonstrated that some metabolic intermediates produced during the tricarboxylic acid cycle, such as citric acid, are inflammatory signals (36), suggesting that there is a greater possibility of inflammation in the LE group than in the ME group, which is consistent with the assessment of inflammation-related gene expression.

### Nucleotide Metabolism

Nucleotides are important components of cells; they are not only involved in the synthesis of genetic material but also play a significant role in energy metabolism, function regulation, and immunity (37). In addition, nucleotides can particularly affect the growth, structure, morphology, and function of the liver. Researchers have demonstrated that exogenous nucleotides or endogenous nucleosides promote the growth of liver cells (38). Uridine, a component of uridine monophosphate (UMP), is naturally produced by the liver. In this study, uridine concentration in the LE, MLE, HE, and MHE groups was significantly lower than that in the ME group, indicating that the liver of the ME group exhibited a better growth potential, which is consistent with the assessment of liver growth provided in Table 1. Furthermore, uridine phosphorylase disrupts hepatic pyrimidine nucleotide metabolism by expressing or inhibiting dihydroorotate dehydrogenase, leading to liver steatosis. Uridine supplementation can inhibit liver steatosis caused by dihydroorotate dehydrogenase (39). The results were consistent with the trend of hepatic steatosis scores provided in Table 2, indicating that the degree of hepatic steatosis may be minimum in the ME group.

### Bile Acid Metabolism

Bile acid is an important component of bile, which is important for digestion and lipid metabolism (40). In recent years, researchers have demonstrated that bile acids affect and regulate physiological processes such as glucolipid metabolism and inflammatory reaction by activating downstream signals through their receptors (41). It has been reported that primary and some secondary bile acids can inhibit the release of TNF, suggesting that bile acids exert an anti-inflammatory effect (42). Therefore, the LE and HE groups were more likely to exhibit an inflammatory reaction, which is consistent with the assessment of inflammation-related gene expression.

### Other Metabolites

Owing to different dietary energy levels, in addition to the above-mentioned liver metabolites, other differential metabolites are related to hepatic health as well, including glutathione, ergothioneine, *p*-cresol, betaine, and cortisol.

Glutathione is a tripeptide of glutamic acid, cysteine, and glycine, which contains γ-amide bonds and sulfhydryl groups. It is involved in converting harmful toxic substances into harmless substances (43, 44), thereby maintaining the normal immune functions of an organism (45). In this study, glutathione concentration was significantly lower in the MLE group than that in the ME group, suggesting that the ME group exhibited better immune function. The results were partially consistent with those of antioxidant analysis because glutathione is a component of GSH-Px.

Ergothioneine is a natural antioxidant with anti-inflammatory and cytoprotective effects (46, 47), which is distributed in certain tissues and organs of mammals. Melville reported that ergothioneine is also found in cereal plants (48). In this study, ergothioneine concentration in the ME group was significantly higher than that in the LE and MLE groups, which may be owing to differences in the intake of maize. However, compared with the ME group, ergothioneine concentration did not increase in the MHE and HE groups, suggesting that its uptake was limited. However, further investigation is required. Owing to the natural antioxidant function of ergothioneine, the antioxidant activity in the ME group was better than that in the LE and MLE groups, which is partially consistent with the results of oxidative stress analysis.

Oxoadipate is metabolized by lysine through the zymocin and pipecolic acid pathways. If lysine, tryptophan, and hydroxylysine are metabolized incorrectly, oxoadipate production is greatly increased (49). In this study, oxoadipate concentration was significantly higher in the LE and HE groups than that in the LME and MHE groups, indicating that low or high dietary energy levels may cause disorders in the lysine, tryptophan, and hydroxylysine metabolism. This is not conducive to liver health.

## Conclusion

In conclusion, based on apparent and molecular evidence, we confirmed that hepatic injury may be induced by lipid accumulation and other altered metabolites. In particular, both high and low dietary energy levels cause hepatic injury in Yunnan semi-fine wool sheep. Based on our research findings, the dietary metabolic energy requirement of Yunnan semi-fine wool sheep is 9.2–9.8 MJ/kg (ME and MHE groups). This study also provides useful information regarding the effect of dietary energy level on the hepatic health of growing sheep at the metabolic level, thereby providing guidance for improving the production efficiency of the sheep.

## Data Availability Statement

The original contributions presented in the study are included in the article/Supplementary Material, further inquiries can be directed to the corresponding author/s.

## Ethics Statement

The animal study was reviewed and approved by the Animal Ethical and Welfare Committee (AEWC) of the Sichuan Agricultural University Academy of Sciences.

## Author Contributions

BCX, QH, and XL designed the studies and prepared the manuscript with comments from all authors. ML, BX, SY, and JZ performed all the experiments and analyzed the data. LW, ZW, and QP revised the manuscript.

## Funding

This research was funded by the National Key Research and Development Program of China (2018YFD0502303) and National Technical System of Wool Sheep Industry (CARS39-08).

## Conflict of Interest

The authors declare that the research was conducted in the absence of any commercial or financial relationships that could be construed as a potential conflict of interest.

## Publisher's Note

All claims expressed in this article are solely those of the authors and do not necessarily represent those of their affiliated organizations, or those of the publisher, the editors and the reviewers. Any product that may be evaluated in this article, or claim that may be made by its manufacturer, is not guaranteed or endorsed by the publisher.

## References

[1] Zhao Y Hong Q Xie P Chen G Wang W. Study on yunnan semi-wool sheep production performance. Acta Ecologiae Animalis Domastici. (2011) 32:51–6. 10.3969/j.issn.1673-1182.2011.06.010

[2] Zhang X Xin W Chen W Zhang Y Zhou Z. Growth performance and development of internal organ, and gastrointestinal tract of calf supplementation with calcium propionate at various stages of growth period. PLoS ONE. (2017) 12:e0179940. 10.1371/journal.pone.0179940 28692656 PMC5503182

[3] Shen Z Syfert H Loehrke B Schneider F Zitnan R Chudy A. An energy-rich diet caused rumen papillae proliferation associated with more IGF type 1 receptors and increased plasma IGF-1 concentration in young goats. J Nutr. (2004) 134:11–17 10.1093/jn/134.1.11 14704286

[4] Noziere P Attaix D Bocquier F Doreau M. Effects of underfeeding and refeeding on weight and cellularity of splanchnic organs in ewes. J Anim Sci. (1999) 77:2279–90. 10.2527/1999.7782279x 10462009

[5] Buhler S Frahm J Tienken R Kersten S Meyer U Huber K. Effects of energy supply and nicotinic acid supplementation on serum anti-oxidative capacity and on expression of oxidative stress-related genes in blood leucocytes of periparturient primi- and pluriparous dairy cows. J Anim Physiol Anim Nutr. (2018) 102:e82–9. 10.1111/jpn.12705 28439984

[6] Clark JM Diehl AM. Nonalcoholic fatty liver disease: an underrecognized cause of cryptogenic cirrhosis. JAMA. (2003) 289:3000–4. 10.1001/jama.289.22.3000 12799409

[7] Tomlinson JW Newsome PN Dowman JK. Pathogenesis of non-alcoholic fatty liver disease. QJM-INT J Med. (2010) 2:103. 10.1093/qjmed/hcp158 19914930 PMC2810391

[8] Bobe G Young J Beitz D. Invited review: pathology, etiology, prevention,and treatment of fatty liver in dairy cows. J Dairy Sci. (2004) 87:3105–24. 10.3168/jds.S0022-0302(04)73446-3 15377589

[9] Reddy JK Sambasiva Rao M. Lipid metabolism and liver inflammation.II.Fatty liver disease and fattyacid oxidation. Am J Physiol-Gastr L. (2006) 290:G852–58. 10.1152/ajpgi.00521.2005 16603729

[10] Jelenik T Rosseisl M Kuda O Jilkova ZM Medrikova D Kus V. AMP—activated proteinkinase o2 subunit is required for the preservation of hepatic in-sulin sensitivity by n-3 polyunsaturated fatty acids. Diabetes. (2010) 59:2737–46. 10.2337/db09-1716 20693347 PMC2963531

[11] Bergeron R Previs SF Cline GW Perret P Russell RR 3rd Young LH. Effect of 5-aminoimi-dazole-4-carboxamide- 1-beta-D- ribofuranoside infusion on invivo glucose and lipid metabolism in lean and obese Zucker rats. Diabetes. (2001) 50:1076–82. 10.2337/diabetes.50.5.1076 11334411

[12] Ringseis R Gessner DK Eder K. Molecular insights into the mechanisms of liver-associated diseases in early-lactating dairy cows: hypothetical role of endoplasmic reticulum stress. J Anim Physiol a Anim Nutr. (2015) 99:626–45. 10.1111/jpn.12263 25319457

[13] Hermier D Salichon MR Guy G Peresson R. Differential channelling of liver lipids in relation to susceptibility to hepatic steatosis in the goose—sciencedirect. Poultry Sci. (1999) 78:1398–406. 10.1093/ps/78.10.1398 10536788

[14] Baraona E Lieber CS. Alcohol and lipids. Recent DevAlcohol. (1998) 14:97–134. 10.1007/0-306-47148-5_5 9751944

[15] Nicholson JK Lindon JC Holmes E. “Metabonomi cs” unders tanding the metabolic responses of living systems to pathophysiological stimuli viamultivariate statistical analysis of biological NMR spectroscopic data. Xenobiotica. (1999) 29:1181–9. 10.1080/004982599238047 10598751

[16] Ippolito DL Lewis JA Yu C Leon LR Stallings JD. Alteration in circulating metabolites during and after heat stress in the conscious rat: potential biomarkers of exposure and organ-specific injury. BMC Physiol. (2014) 14:14. 10.1186/s12899-014-0014-0 25623799 PMC4306243

[17] Zhang J Wang G Zhao C. 1H NMR plasma metabolomic profiling of ovarianquiescence in energy balanced postpartum dairy cows. Vet Quart. (2018) 38:47–52. 10.1080/01652176.2018.1473660 29733756 PMC6830969

[18] Li X Lu ML Hong QH Xue B Wu YH Hu AH. Energy requirement of yunnan semi-fine-wool sheep during growing period by regression model method. Acta Zoonutr Sin. (2020) 32:447–54. 10.3969/j.issn.1006-267x.2020.01.052

[19] Fujiang W Ruiyan L Pengfei T Jianping C Kewu Z Yong J. Total glycosides of cistanche deserticola promote neurological function recovery by inducing neurovascular regeneration via Nrf2/Keap-1 pathway in MCAO/R Rats. Front Pharmacol. (2020) 11:236. 10.3389/fphar.2020.00236 32256351 PMC7089931

[20] Cao Z Xia W Zhang X Yuan H Gao L. Hepatotoxicity of nutmeg: a pilot study based on metabolomics. Biomed Pharmacother. (2020) 131:110780. 10.1016/j.biopha.2020.110780 33152938

[21] Chilliard Y F Bocquier M Doreau. Digestive and metabolic adaptations of ruminants to undernutrition, and consequences on reproduction. A review. Reprod Nutr Dev. (1998) 38:129–150. 10.1051/rnd:19980201 9638788

[22] Kamalzadeh A Koops W Bruchem J Tamminga S Zwart D. Feed quality restriction and compensatory growth in growing sheep: development of body organs. Small Ruminant Res. (1998) 29:71–82. 10.1016/S0921-4488(97)00111-9

[23] Wang X Huang B Li X Wang S Cheng D. Antagonistic effect of tea polyphenols on erythrocyte and liver injury in acute cadmium (ll) exposure for mice. J Chin Inst Food Sci Tech. (2020) 20:66–72. 10.16429/j.1009-7848.2020.04.009

[24] Dandona P Aljada A Bandyopadhyay A. Inflammation: the link between insulin resistance, and obesity and diabetes. Trends Immunol. (2004) 25:4–7. 10.1016/j.it.2003.10.013 14698276

[25] Li S Khafipour E Krause DO Kroeker A Rodriguez-Lecompte JC Gozho GN. Effects of subacute ruminal acidosis challenges onfermentation and endotoxins in the rumen and hindgut of dairy cows. J. Dairy Sci. (2012) 95:294–303. 10.3168/jds.2011-4447 22192209

[26] Sozio MS Liangpunsakul S Crabb D. The role of lipid metabolism in the pathogenesis of alcoholic and nonalcoholic hepatic steatosis. Semin Liver Dis. (2010) 30:378–90 10.1055/s-0030-1267538 20960377

[27] Grummer RR. Etiology of lipid-related metabolic disorders in periparturient dairy cows. J Dairy Sci. (1993) 76:82–96. 10.3168/jds.S0022-0302(93)77729-2 8132893

[28] Xue BC Zhang JX Wang ZS Wang LZ Peng QH Da LC. Metabolism response of grazing yak to dietary concentrate supplementation in warm season. Animal. (2021) 15:100175. 10.1016/j.animal.2021.100175 33610519

[29] Jiang S Zhang L Zhang HA. novel miR-155/miR-143 cascade controls glycolysis by regulating hexokinase 2 in breast cancer cells. EMBO Jl. (2012) 31:1985–98. 10.1038/emboj.2012.45 22354042 PMC3343331

[30] Wu GY Gunasekara A Brunengraber H. Effects of extracellular pH, CO2, and HCO3-on ketogenesis in perfused rat liver. Am J Physiol-Gastr L. (1991) 261:E221–6. 10.1152/ajpendo.1991.261.2.E221 1908188

[31] Wang H Niu W Wu F Qiu X Yu Z He Y. Effects of dietary energy on antioxidant capacity, glucose–lipid metabolism and meat fatty acid profile of Holstein bulls at different ages. J Anim Physiol Anim Nutr. (2020) 105:1–11. 10.1111/jpn.13457 33006191

[32] Gao L Wu J Song S Li H Lang X Wei Y. Effect of different energy levels on serum lipid index and lipid deposition of Altay sheep. Feed Ind. (2020) 41:14–23. 10.13302/j.cnki.fi.2020.09.003

[33] Wu Z Li M Zhao C. Urinary metabonomics study in a rat model in response to protein-energy malnutrition by using gas chromatography-mass spectrometry and liquid chromatography-mass spectrometry. Mol Biosyst. (2010) 6:2157–63. 10.1039/c005291d 20717558

[34] Li D Lun Y Zhou S. Recent Progress of NAD+/NADH Metabolism. Lett Biotechnol. (2010) 21:98–102.

[35] Lu Y Tang X Li B Ge Y Yang S Zhang K. Mechanism of food-borne tyrosine oxidation product-induced myocardial oxidative damage and energy metabolism disorder in mice. Food Science, (2020) 41:84–90. 10.7506/spkx1002-6630-20191018-180

[36] Mills E O'Neill LA. Succinate: a metabolic signal in inflammation. Trends Cell Biol. (2013) 11:127–31. 10.1016/j.tcb.2013.11.008 24361092

[37] Li B Wu X Zhang B Yin Y. Research progresses on nutrition metabolism and physiological function of uridine monophosphate. Acta Zoonutr Sin. (2019) 31:2487–94. 10.3969/j.issn.1006-267x.2019.06.006

[38] Torres-Lopez MI Fernandez I Fontana L Gil A Rios A. Influence of dietary nucleotides on liver structural recovery and hepatocyte binuclearity in cirrhosis induced by thioacetamide. Gut. (1996) 38:260–4. 10.1136/gut.38.2.260 8801208 PMC1383034

[39] Le TT Ziemba A Urasaki Y Hayes E Pizzorno G. Disruption of uridine homeostasis links liver pyrimidine metabolism to lipid accumulation. J Lipid Res. (2013) 54:1044. 10.1194/jlr.M034249 23355744 PMC3605981

[40] Chavez-Talavera O Wargny M Pichelin M Descat A Vallez E Kouach M. Bile acids associate with glucose metabolism,but do not predict conversion from impaired fasting glucose to diabetes. Metabolism. (2020) 103:154042. 10.1016/j.metabol.2019.154042 31785259

[41] Perino A Schoonjans K. TGR5 and immunometabolism: insights from physiology and pharmacology. Trends Pharmacol Sci. (2015) 36:847–57. 10.1016/j.tips.2015.08.002 26541439

[42] Qi M Diao Q Zhang N. Advance in ruminal development and its influencing factors in lambs. Chin J Anim Sci. (2015) 51:77–81. 10.3969/j.issn.0258-7033.2015.09.017

[43] Treviani F Tame MR. Sovere hep-atic failureoeeurring with T6l ingestion in an at-tempted suieide Earlyreeovery with the use of intra-venous infusion of redueedglutathione. Dig Dis Sci. (1993) 38:752–6. 10.1007/BF01316810 8462375

[44] Morel G Bonnet P Cossee B. The role of glu-tathione andcysteine conjugate in the nephrotoxieity of o-xylene in rats. Arehtoxicol. (1998) 72:553–8. 10.1007/s002040050542 9806426

[45] Kashiwagi A Asahina A Tkebuehi M. Abnomal glutathione metabolism and inereased eytotoxieity caused by H2O2: inhuman umbilical vein endothelial cells cultured in high glueosemedium. Diabetologia. (1994) 37:264–9. 10.1007/BF00398053 8174840

[46] Yoshida S Shime H Funami K Takaki O Matsumoto M Kasahara M. The anti-oxidant ergothioneine augments theimmunomodulatory function of TLR agonists by direct action on macrophages. Plos One. (2017) 12:1–15. 10.1371/journal.pone.0169360 28114402 PMC5256913

[47] Cheah IK Halliwell B. Ergothioneine; antioxidant potential, physiology function and role in disease. Biochim Biophys Acta. (2012) 1822:784–93. 10.1016/j.bbadis.2011.09.017 22001064

[48] Melville DB Eich S. The occurrence of ergothioneine in plante meterial. J Biol Chem. (1956) 218:647–51. 10.1016/S0021-9258(18)65831-4 13295219

[49] Luo Z Bai L Huang Y Jiang J Shen L Tao J. Effect of selenium yeast on plasma metabolism of transition dairy cow in parturition stress status. J Northwest A & F Univ. (2019) 49:7–15. 10.13207/j.cnki.jnwafu.2019.02.002

