# Supplementary material for: Hepatic Injury Induced by Dietary Energy Level via Lipid Accumulation and Changed Metabolites in Growing Semi-Fine Wool Sheep
Source: Front Vet Sci. 2021 Sep 23;8:745078. doi: 10.3389/fvets.2021.745078 (PMC8494768; doi:10.3389/fvets.2021.745078)
Supplement: Supplementary file 1 [file Data_Sheet_1.docx]

**Supplementary Materials**

**Table S1**. Primer sequences for Real-time PCR

| Genes | Accession No. | Primes（5'-3'） |
| --- | --- | --- |
| IL-1β | NM_001009465.2 | F: GAAGAGCTGCACCCAACACCTG |
|  |  | R: CGACACTGCCTGCCTGAAGC |
| IL-6 | NM_001009392.1 | F: GCTGCTCCTGGTGATGACTTCTG |
|  |  | R: AGTAGTCTGCTTGGGGTGGTGTC |
| NFKB | XM_027960471.1 | F: GCCTGCTGAATGCCCTGTCTG |
|  |  | R: CTCTGTTTCCTGTTCCACCGACTG |
| NOD-2 | XM_015100434.2 | F: TGCCATCCTCGCTCAGACATCTC |
|  |  | R: CAGCCACACTGCCCTCTTTGC |
| TLR-2 | NM_001048231.1 | F: TGCCCGCCTCTCCCTTTCTG  R: CGTGAGCAGGAGCAACAGGAAG |
| TLR-3 | NM_001135928.1 | F: ACACAATCAGCCACACGACCTTC  R: GCCAGGCAAAGGAGTCATTACCC |
| TLR-4 | NM_001135930.1 | F: TGGGTGCGGAATGAACTGGTAAA  R: CTGGATGATATTGGCGGCGATGG |
| TNF-α | NM_001024860.1 | F: CTGGCGGAGGAGGTGCTCTC  R: GGAGGAAGGAGAAGAGGCTGAGG |
| GAPDH | NM_001190390.1 | F: CGGCACAGTCAAGGCAGAGAAC  R: CACGTACTCAGCACCAGCATCAC |

*IL-1β* interleukin-1β, *IL-6* interleukin-1β, *NFKB* nuclear factor-k-gene binding, *NOD2* nucleotide-binding oligomerization domain, *TLR2* Toll-like receptors2, *TLR3* Toll-like receptors3, *TLR4* Toll-like receptors4, *TNF-α* tumor necrosis factor alpha, *GAPDH* glyceraldehyde-3-phosphate dehydrogenase..


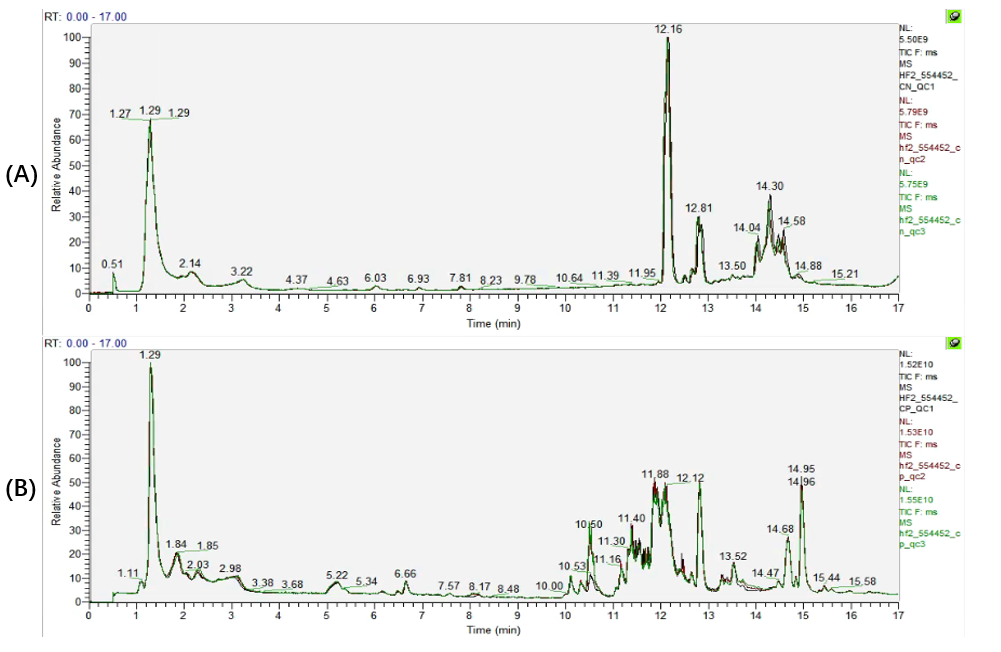


**Figure S1**. Total Ion Chromatography of Quality control samples in positive (A) and negative (B) modes.


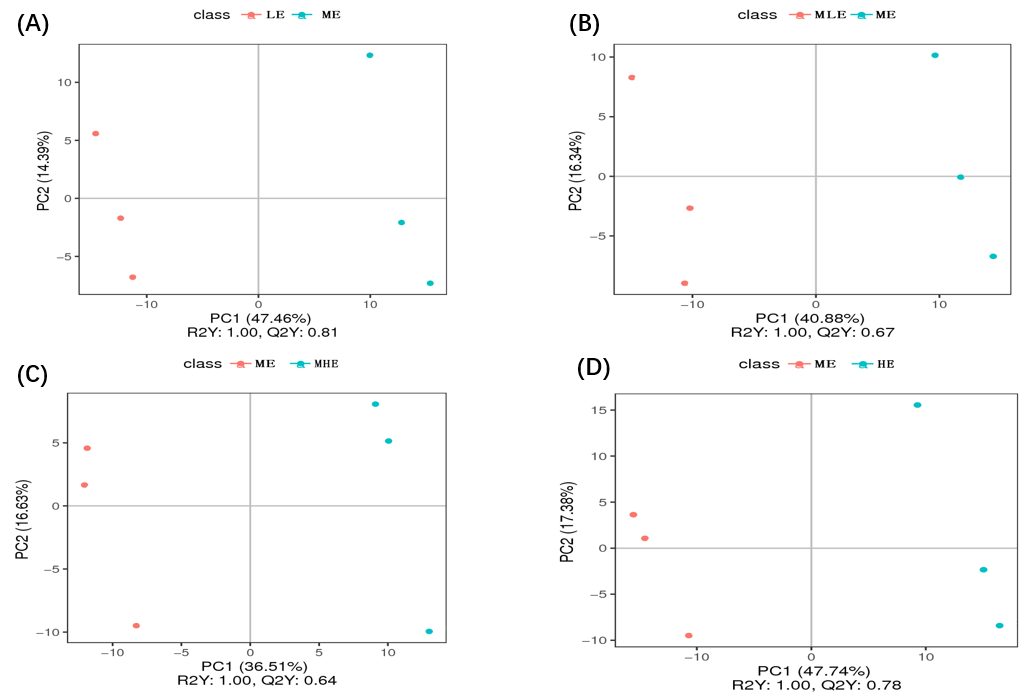


**Figure S2**. . PLS-DA loading plots based on the liver metabolic profilings in positive mode. A: LE Group VS ME group. B: MLE Group VS ME group. C: MHE Group VS ME group. D: HE Group VS ME group.


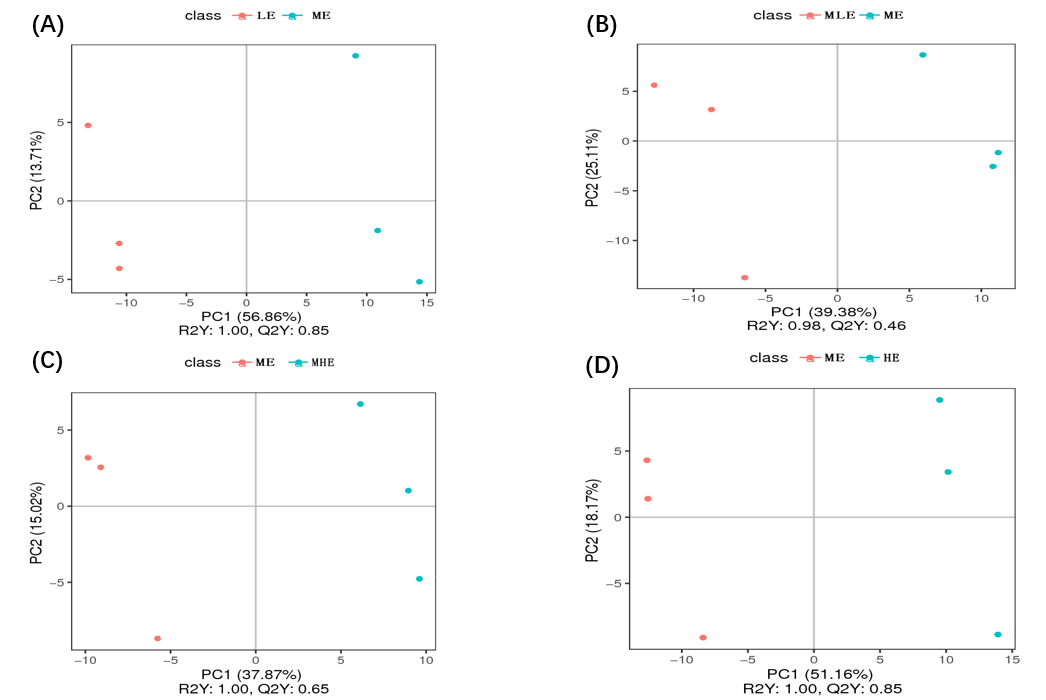


**Figure S3**. PLS-DA loading plots based on the liver metabolic profilings in negative mode. A: LE Group VS ME group. B: MLE Group VS ME group. C: MHE Group VS ME group. D: HE Group VS ME group.
